# S‐Nitrosylation of NOTCH1 Regulates Mesenchymal Stem Cells Differentiation Into Hepatocyte‐Like Cells by Inhibiting Notch Signalling Pathway

**DOI:** 10.1111/jcmm.70274

**Published:** 2024-12-10

**Authors:** Xuesong Wang, Yan Xu, Yue Wang, Xingkun Tang, Xiaolei Zhou, Wenming Lu, Wenjie Chen, Lincai Li, Lin Zhou, Junsong Ye

**Affiliations:** ^1^ Subcenter for Stem Cell Clinical Translation First Affiliated Hospital of Gannan Medical University Ganzhou Jiangxi China; ^2^ School of Rehabilitation Medicine Gannan Medical University Ganzhou Jiangxi China; ^3^ Ganzhou Key Laboratory of Stem Cell and Regenerative Medicine Ganzhou Jiangxi China; ^4^ College of Nursing, Gannan Medical University Ganzhou Jiangxi China; ^5^ Jiangxi Provincial Key Laboratory of Tissue Engineering Gannan Medical University Ganzhou Jiangxi China; ^6^ Key Laboratory of Prevention and Treatment of Cardiovascular and Cerebrovascular Diseases Ministry of Education, Gannan Medical University Ganzhou Jiangxi China

**Keywords:** differentiation mechanisms, hepatogenic differentiation, mesenchymal stem cells, NO, signalling pathways, S‐nitrosylation

## Abstract

The differentiation of mesenchymal stem cells (MSCs) into hepatocyte‐like cells (HLCs) is considered one of the most promising strategies for alternative hepatocyte transplantation to treat end‐stage liver disease. To advance this method, it is crucial to gain a deeper understanding of the mechanisms governing hepatogenic differentiation. The study demonstrated that suppression of the intracellular domain release of the Notch pathway receptor via the γ‐secretase inhibitor N‐[(3, 5‐difluorophenyl)acetyl]‐L‐alanyl‐2‐phenylglycine‐1, 1‐dimethylethyl ester (DAPT) significantly promotes the expression of hepatocyte‐related genes and proteins in HLCs. Increased expression of intracellular inducible NO synthase (iNOS) during differentiation led to elevated endogenous NO production. Biotin switch assays revealed a gradual increase in S‐nitrosylation (SNO)‐NOTCH1 and a decrease in overall NOTCH1 expression during hepatogenic differentiation. The addition of the exogenous NO donor S‐nitrosoglutathione (GSNO) and the SNO inhibitor dithiothreitol (DTT) further demonstrated that the elevated expression of SNO‐NOTCH1 promotes the differentiation of MSCs into mature hepatocytes. Briefly, our results fully demonstrated that the modification of the extracellular domain of NOTCH1 by NO, leading to the formation of SNO‐NOTCH1, significantly promotes hepatogenic differentiation by inhibiting the Notch signalling pathway. Our study highlights the critical role of SNO‐NOTCH1 in regulating the Notch signalling pathway and offers new insights into the mechanisms driving this differentiation process.

**Abbreviations**
AFP: alpha‐fetoprotein
ALB: albumin
BLVRB: recombinant biliverdin reductase B
CK‐18: cytokeratin‐18
CK‐19: cytokeratin‐19
Dex: dexamethasone
EGF: epidermal growth factor
ESCs: embryonic stem cells
FGF4: basic fibroblast growth factor
HGF: hepatocyte growth factor
HLCs: hepatocyte‐like cells
HNF4α: hepatocyte nuclear factor 4α
HT: hepatocyte transplantation
hUCMSCs: human umbilical cord mesenchymal stromal hepatocytes
iPSCs: induced pluripotent stem cells
LT: liver transplantation
MSCs: mesenchymal stem cells
NECD: Notch extracellular receptor domain
NICD: Notch intracellular receptor domain
OSM: oncostatin M
SNO: S‐nitrosylation

## 1 Introduction

The liver is an important organ for drug metabolism and detoxification in the human body. Currently, liver transplantation (LT) and hepatocyte transplantation (HT) are considered the most effective treatments for severe liver failure, hepatocellular carcinoma and other end‐stage liver diseases [1, 2]. However, LT faces challenges such as a shortage of donor livers, immune rejection and high costs [3], while HT has not been widely adopted in clinical practice due to issues like unclear hepatocyte sources, high cell demand and the limited ability of in vitro cells to self‐proliferate [4]. In recent years, researches have indicated that HLCs differentiated from MSCs possess the functions of hepatocytes in vitro, positioning them as a promising alternative to LT and HT for end‐stage liver disease [5, 6]. Human umbilical cord mesenchymal stem cells (hUCMSCs) offer advantages such as ease of acquisition, wide availability, multidirectional differentiation potential, low immune rejection and minimal ethical concerns. Therefore, hUCMSCs are increasingly utilised for targeting differentiation into HLCs and are considered ideal seed cells for the treatment of end‐stage liver disease and liver regenerative medicine [7, 8]. A deeper exploration of the mechanisms underlying the differentiation of MSCs into HLCs could accelerate their clinical application in treating end‐stage liver disease. The Notch signalling pathway, a conserved signalling pathway, plays an important role in regulating MSC differentiation [9, 10, 11]. Previous studies have shown that Notch signalling pathway activity decreases during the hepatogenic differentiation of MSCs [12]. Additionally, the differentiation of bone marrow‐derived MSCs into hepatocytes is facilitated by the increased expression of the Notch pathway negative regulator Numb [13]. Although it is evident that the Notch signalling pathway affects hepatogenic differentiation of MSCs, the exact mechanisms by which it regulates this process are not yet fully understood.

S‐nitrosylation, a post‐translational modification mediated by NO on cysteine sulfhydryl groups, influences a variety of biological processes and signalling pathways. It modulates protein biochemical activities, stability, subcellular localization and protein–protein interactions, among other effects. Evidence indicates that S‐nitrosylation significantly impacts cell differentiation and maturation by altering gene transcription [14], enzyme activity [15] and nucleoprotein translocation [16]. Research has shown that NO can modulate the expression of the Notch signalling pathway receptor NOTCH1 [17, 18], which is crucial for signal transduction [19, 20]. However, the detailed mechanisms by which NO regulates NOTCH1, its potential role in directing the hepatogenic differentiation of MSCs and how NO influences the maturation of HLCs are still to be determined.

In this study, the γ‐secretase inhibitor DAPT was incorporated into the differentiation process to inhibit the Notch signalling pathway by preventing its third cleavage. Comparative analysis of the maturation levels of hepatocyte‐associated factors following the inhibition of the Notch signalling pathway revealed that this pathway plays a regulatory role in the differentiation of MSCs into HLCs. We also confirmed that increased levels of SNO‐NOTCH1 were attributed to elevated NO levels resulting from the positive expression of iNOS. To deepen our understanding of the impact of SNO‐NOTCH1 on hepatogenic differentiation, we modulated the level of SNO‐NOTCH1 by introducing GSNO, an exogenous NO donor capable of stable NO release, and DTT, which reduces the sulfhydryl groups of proteins involved in S‐nitrosylation, during hepatogenic differentiation. Our findings indicate that SNO‐NOTCH1 exerts a critical regulatory role during the directional differentiation of MSCs. Our research provides both theoretical and experimental insight into the mechanism of HLCs’ formation, facilitating the clinical translation of MSCs‐derived HLCs and offering new possibilities for the treatment of liver diseases.

## 2 Materials and Methods

### 2.1 Isolation and Culture of MSCs

MSCs were derived from the umbilical cords of healthy women at the First Affiliated Hospital of Gannan Medical University. Prior to collection, the umbilical cord donors provided written informed consents, acknowledging that the umbilical cords would be used exclusively for subsequent research. The collected umbilical cord was initially sterilised using 75% ethanol by volume and then rinsed with sterile PBS (Gibco, USA). Subsequently, it was sectioned into 3–5 cm segments using scissors, and the blood vessels were isolated following longitudinal dissection of each segment. Erythrocytes and endothelial cells were removed through washing with pre‐cooled PBS. The cord segments were then distributed evenly in culture dishes (Bioland, China) and incubated at 37°C with 5% CO₂ to facilitate cell isolation and growth. The culture medium employed was DMEM‐LG (Gibco, USA), supplemented with 10% foetal bovine serum (FBS) (Gibco, New Zealand). Cells were passaged upon reaching approximately 70% confluency, detached using 0.25% trypsin (Biochem, China) and replanted in appropriate culture dishes.

To ensure that the isolated cells meet the standards of MSCs, it is essential to standardise the quality identification process [21]. Consistent with our previous study [22], MSC identification was mainly performed using osteogenic (Cyagen, China) and adipogenic (Cyagen, China) induction solutions. These solutions induce the differentiation of MSCs into osteoblasts or adipocytes to assess their multilineage differentiation potential. Moreover, flow cytometry (BD, USA) was employed for the quantitative analysis of cell surface markers, including CD44, CD90, CD105, CD34, CD45 and HLA‐DR (Invitrogen, USA).

### 2.2 Hepatogenic Differentiation Procedure of MSCs and Characterisation of HLCs

Induction experiments were conducted on passage 3 (P3) MSCs with optimal growth conditions. A total of 1*10^5^ MSCs were inoculated per well into 6‐well plates (Bioland, China). When the cells reached 80% confluence, the hepatogenic differentiation induction solution was added in the following order: Serum‐free culture medium: DMEM‐LG + 20 μg/L Epidermal growth factor (EGF) (Glpbio, USA) + 10 μg/L Basic fibroblast growth factor (FGF4) (Proteintech, USA) for 2 days; Differentiation medium: DMEM‐LG + 20 μg/L Hepatocyte growth factor (HGF) (Proteintech, USA) + 10 μg/L FGF4 + 0.61 g/L Nicotinamide (Sigma‐Aldrich, USA) for 7 days with culture medium changes every 3 days; Maturation medium: DMEM‐LG + 20 μg/L Oncostatin M (OSM) (Proteintech, USA) + 1 μmol/L Dexamethasone (Dex) (Glpbio, USA) + 1 × ITS premix (Gibco, USA), induced for 28 days with medium changes every 3 days.

To validate the functional characterisation of HLCs, we assessed the glycogen synthesis capability of differentiated HLCs after 28 days using periodic acid‐Schiff (PAS) staining (Beyotime, China). Briefly, HLCs were fixed in anhydrous ethanol, oxidised with periodic acid solution for 10 min and stained with Schiff's reagent for 45 min. Finally, the nuclei were stained with haematoxylin for 10 min. In addition, the low‐density lipoprotein (LDL) uptake capacity of HLCs was evaluated using the Dil‐LDL Staining Kit (Solarbio, China), following exactly the manufacturer's instructions. The indocyanine green (ICG) uptake ability of HLCs was further characterised using ICG reagent (Sigma, USA), and the cells were stained and observed under an inverted microscope (Zeiss, Germany).

### 2.3 Quantitative Real‐Time PCR Analysis

Total RNA was extracted from cells using Trizol (TransGen Biotech, China). After quantifying the nucleic acid, reverse transcription (TransGen Biotech, China) was performed. Real‐time quantitative PCR analyses were then conducted using SYBR Green Supermix (TransGen Biotech, China) and analysed with a system (Bio‐rad, USA). The relative expression levels of each gene were calculated using the 2^−ΔΔCt^ method, with GAPDH as the internal reference. Specific primers (Generay Biotech, China) used are listed in Table S1.

### 2.4 Transcriptome Sequencing

After 28 days of differentiation, total RNA was extracted from the cells using the RNeasy Plus Mini Kit (Qiagen). Sequencing data were provided by Guangzhou Gene Denovo RNA, and the quantity and integrity of the RNA were verified by agarose gel electrophoresis, NanoDrop microspectrophotometer and Agilent 2100 Bioanalyzer. The RNA‐seq library was constructed using Illumina's NEBNext Ultra RNA Library Preparation Kit according to the manufacturer's protocol. The library was then assessed using the Agilent 2100 Bioanalyzer. Finally, 150 bp paired‐end sequencing was performed on the Illumina NovaSeq 6000 HiSeq sequencer. The raw data from Illumina HiSeq sequencing underwent quality control processing, and the resulting clean reads were used for downstream bioinformatics analysis. HISAT2 software was used to align the clean reads to the reference genome, and gene expression levels were determined by calculating the fragments per kilobase of transcript per million mapped reads (FPKM). Differentially expressed genes (DEGs) were identified using the DESeq R package, with the criteria |log_2_ fold change| > 1 and *p* < 0.05. Differential expression analysis was conducted using the Omicamart website (https://www.omicsmart.com), and Fisher's exact test was applied to identify significantly enriched Gene Ontology (GO) terms and significant enrichment pathways associated with DEGs, with *p* < 0.05 considered statistically significant.

### 2.5 Cytotoxicity Assay

In this study, we evaluated the cytotoxicity of DAPT, GSNO and DTT (all from Glpbio, USA) on MSCs using the Cell Counting Kit‐8 (CCK‐8) assay (Apexbio, USA). MSCs were seeded in 96‐well cell culture plates at a density of 5 × 10^4^ cells per well. Upon reaching 80% confluence, the cells were exposed to varying concentrations of DAPT, GSNO and DTT, with each concentration replicated across eight wells. Cells that received no treatment served as negative controls. Following a 24‐h incubation period, cell biocompatibility was assessed using CCK‐8, with optical density measurements taken at 450 nm, following the manufacturer's protocol.

### 2.6 Cell Cycle Analysis of MSCs

MSCs were seeded at a density of 0.2 × 10^6^ cells per 25 cm^2^ in culture flasks. Upon achieving 80% confluence, the cells were trypsinised. Hepatogenic differentiation was initiated both with and without the addition of DAPT. Twenty‐four hours post‐differentiation, the cells underwent another round of trypsinisation. Subsequently, these cells were washed with PBS and fixed in 70% ethanol at 4°C. The cells were treated with 0.5 mg/mL RNase A (BD, USA) and stained with 50 μg/mL propidium iodide (BD, USA) in the dark for 30 min at 37°C. DNA content was quantified by flow cytometry, assessing the percentage of cells in various phases of the cell cycle. A minimum of 10,000 cells were analysed using flow cytometry, and the data were further processed using Modfit LT 5.0 software.

### 2.7 Western Blotting

Cells were lysed using RIPA buffer (EpiZyme, China) with added protease inhibitors to extract total protein from each cell group. Proteins were separated by SDS‐PAGE with a concentration of 8% to 15% (depending on the molecular weight of the proteins) and then transferred to a PVDF membrane (Millipore, USA). The membrane was blocked with TBST buffer containing 5% bovine serum albumin (EpiZyme, China) at room temperature for 1 h. The membrane was then incubated overnight at 4°C with primary antibodies, including anti‐NOTCH1 antibody (Abcam, UK), anti‐NICD antibody (Proteintech, USA), anti‐HES1 antibody (Abcam, UK), anti‐ALB antibody (Proteintech, USA), anti‐AFP antibody (Proteintech, USA), anti‐HNF4α antibody (Proteintech, USA), anti‐CK‐19 antibody (Proteintech, USA), anti‐iNOS antibody (Proteintech, USA), anti‐eNOS antibody (Proteintech, USA), anti‐nNOS antibody (Proteintech, USA) and anti‐GAPDH antibody (Proteintech, USA). Following this, the membrane was incubated at room temperature for 1 h with goat anti‐rabbit IgG secondary antibody (Proteintech, USA) conjugated with horseradish peroxidase, diluted 1:5000. Finally, the membrane was visualised using an enhanced chemiluminescence system (Bio‐Red, USA), and the grey values of the images were analysed using Image Lab software.

### 2.8 Biotin Switch Assay of S‐Nitrosylation

As described in our previous study [23], the biotin switch assay was utilised to detect specific proteins in cells. Briefly, cells were lysed using HENS buffer and then blocked with a blocking buffer for 30 min to block free sulfhydryl groups. Proteins were precipitated with cold acetone at the bottom of a centrifuge tube. Subsequently, S‐nitrosothiols in the proteins were reduced to free thiols by vitamin C and biotinylated using a labelling buffer. Biotin was adsorbed by streptavidin‐agarose gels, and the adsorbed sulfhydryl groups were subsequently eluted through denaturation [24]. Finally, SDS‐PAGE analysis was performed using NOTCH1 or NICD antibodies.

### 2.9 Measurement of NO Levels

At the differentiation time points of 7, 14 and 28 days for MSCs, intracellular NO was fluorescently labelled with diaminofluorescein‐FM diacetate (DAF‐FM‐DA) (TargetMol, USA) for 20 min. The cells were then washed three times with PBS to remove any residual fluorescent probes. Images were captured using a fluorescence microscope (Zeiss, Germany). The exact NO content was measured using the Grise reagent (Beyotime, China) method. Briefly, standards were diluted with DMEM, and both standards and samples were added separately to a 96‐well plate. Griess reagent I and Griess reagent II were then mixed, and the absorbance was measured at 540 nm to determine the NO content.

### 2.10 Grouping Experiment

Effect of the Notch signalling pathway on the directed differentiation of MSCs into HLCs in vitro:

The separated P3 MSCs were divided into three groups for cultivation: ① Undifferentiation (Un‐Diff) group: MSCs were cultured in DMEM medium supplemented with 10% FBS; ② Hepatogenic differentiation (Hep‐Diff) group: When the cell confluence reached 90%, DMEM containing inducing factors was added into the medium to initiate hepatogenic differentiation; ③ DAPT group: Following the differentiation protocol of the Hep‐Diff group, added DAPT, a Notch signalling pathway inhibitor, at a final concentration of 10 μM at each change of cell culture medium for treatment from day 1 until day 28 [25].

Effect of SNO‐NOTCH1 on the Notch signalling pathway and hepatic induction of differentiation in vitro:

The separated P3 MSCs were divided into four groups for cultivation: ① Undifferentiation (Un‐Diff) group: MSCs were cultured in DMEM medium supplemented with 10% FBS; ② Hepatogenic differentiation (Hep‐Diff) group: When the cell confluence reached 90%, DMEM containing inducing factors was added into the medium to initiate hepatogenic differentiation; ③ GSNO group: Following the differentiation protocol of the Hep‐Diff group, GSNO, an exogenous NO donor, at a final concentration of 10 μM at each change of cell culture medium for treatment from day 1 until day 28 [26]; ④ DTT group: Following the differentiation protocol of the Hep‐Diff group, DTT, an S‐nitrosylation modification inhibitor, at a final concentration of 10 μM at each change of cell culture medium for treatment from day 1 until day 28 [27].

### 2.11 Statistical Analysis

All data were expressed as mean ± standard deviation (SD) and were statistically analysed using GraphPad Prism 10.0 or SPSS 20.0. Comparisons between groups were conducted using unpaired *t*‐tests or one‐way ANOVA. Each data point represented the result of at least three independent experiments. Differences were considered statistically significant at *p* < 0.05.

## 3 Results

### 3.1 Differentiation of MSCs and Characterisation of HLCs

**FIGURE 1:**
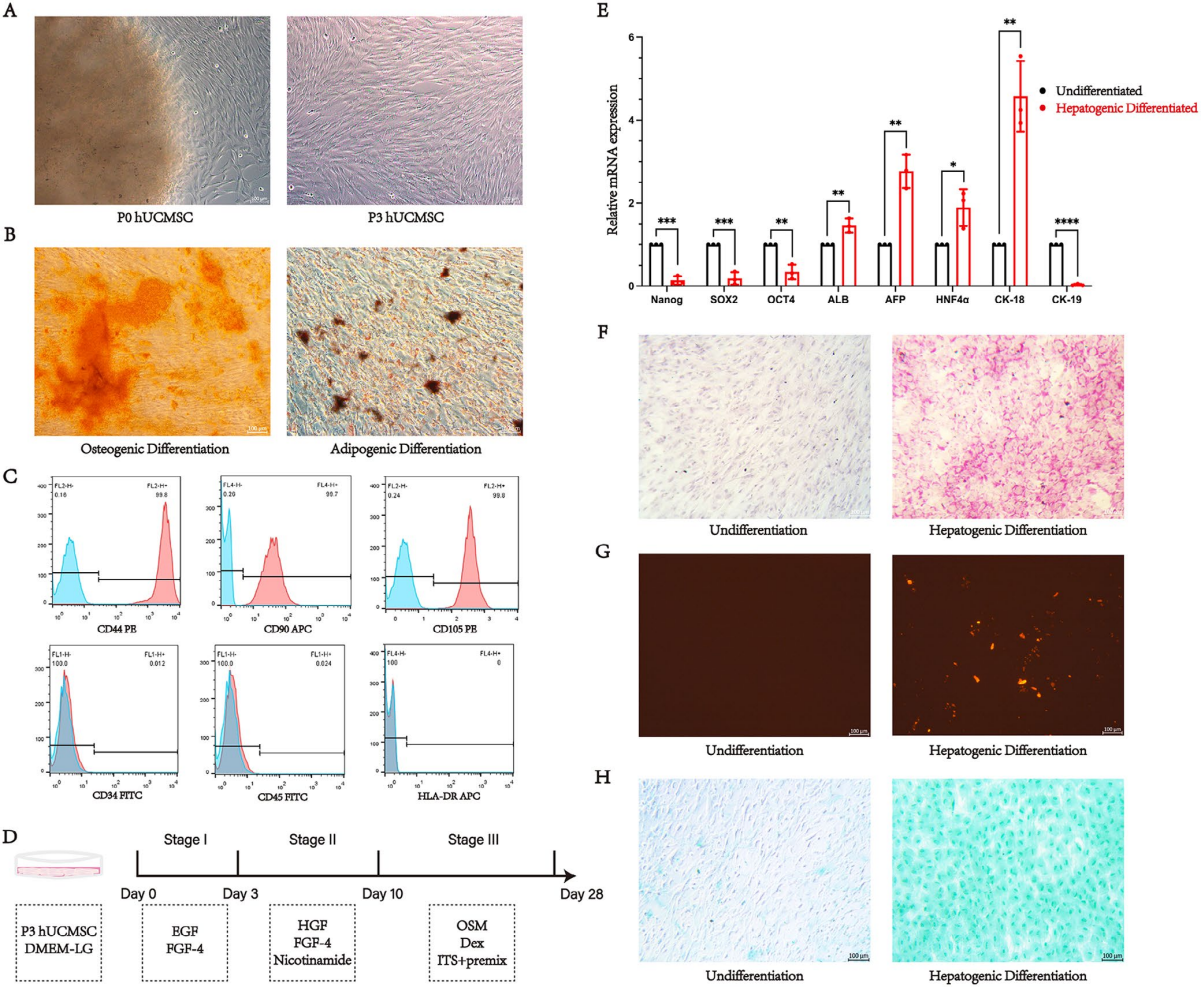
The characteristics of MSCs and their differentiation into functional HLCs in vitro. (A). Morphological characteristics of MSCs at P0 and P3. Scale bar = 100 μm. (B) Demonstration of the osteogenic and adipogenic differentiation potential of MSCs by Alizarin red staining and Oil Red O staining. Scale bar = 100 μm. (C) Expression of cell surface markers on MSCs. Cells were incubated with FITC, PE or APC‐conjugated anti‐human‐specific antibodies. Flow cytometry analysis revealed positive expression of CD44, CD90 and CD105, while CD34, CD45 and HLA‐DR were negative. (D) Induction protocol for differentiation of MSCs into HLCs. EGF. Epidermal growth factor; FGF4, Basic fibroblast growth factor; HGF, Hepatocyte growth factor; OSM, Oncostatin M; Dex, Dexamethasone. (E) RT‐qPCR analysis of mRNA expression levels of pluripotent transcription factors (NANOG, SOX2 and OCT4) and hepatocyte‐associated factors (ALB, AFP, HNF4α, CK‐18 and CK‐19) in the Un‐Diff group and the Hep‐Diff group. *P < 0.05, **P < 0.01, ***P < 0.001. (F) PAS staining demonstrating glycogen synthesis in Un‐Diff group and Hep‐Diff group. Scale bar = 100 μm. (G) Dil‐LDL labelling showing LDL uptake capacity of cells in Un‐Diff group and Hep‐Diff group. Scale bar = 100 μm. (H) ICG reagent demonstrating ICG uptake capacity of cells in Un‐Diff group and Hep‐Diff group. Scale bar, 100 μm.

The MSCs were isolated using the tissue explant adherent method by culturing umbilical cord tissue in a medium containing 10% FBS for 1 week. Subsequently, the cells were digested and passaged to P3 to obtain purified MSCs (Figure 1A). In vitro osteogenic and adipogenic differentiation experiments were then conducted on these cells. At 21 days of induction, cells were stained with Alizarin red and Oil Red O reagents, respectively, confirming the multilineage differentiation ability of MSCs (Figure 1B). Flow cytometry was used to detect molecular markers of P3 cells, revealing that the cell surface markers CD44 (99.8%), CD90 (99.7%) and CD105 (99.8%) were positively expressed, whereas CD34 (0.012%), CD45 (0.024%) and HLA‐DR (0%) were negatively expressed (Figure 1C). These results confirm that the isolated cells possess high‐purity MSC characteristics.

P3 MSCs underwent the hepatogenic differentiation cytokine induction method (Figure 1D). mRNA analysis revealed that after 28 days of induction, the expression levels of stem cell pluripotent transcription factors, NANOG, SOX2 and OCT4, were significantly downregulated. Conversely, the mRNA expression levels of hepatocyte‐associated factors ALB, AFP, HNF4α and CK‐18 increased, while CK‐19 mRNA expression decreased (Figure 1E). The expression of ALB, AFP, HNF4α and CK‐18 was observed to be positive, while CK‐19 was negative in mature hepatocytes [28]. These findings suggest that during hepatogenic differentiation, MSCs progressively lose their stem cell characteristics and transition towards mature HLCs.

Additionally, PAS staining, LDL uptake assay and ICG uptake assay were performed on the induced HLCs. The results demonstrated the functional resemblance of HLCs differentiated from MSCs to mature hepatocytes (Figure 1F–H). These findings further indicate that MSCs have successfully differentiated into functional HLCs in vitro.

### 3.2 Changes in Key Genes of the Notch Signalling Pathway After Hepatogenic Differentiation

**FIGURE 2:**
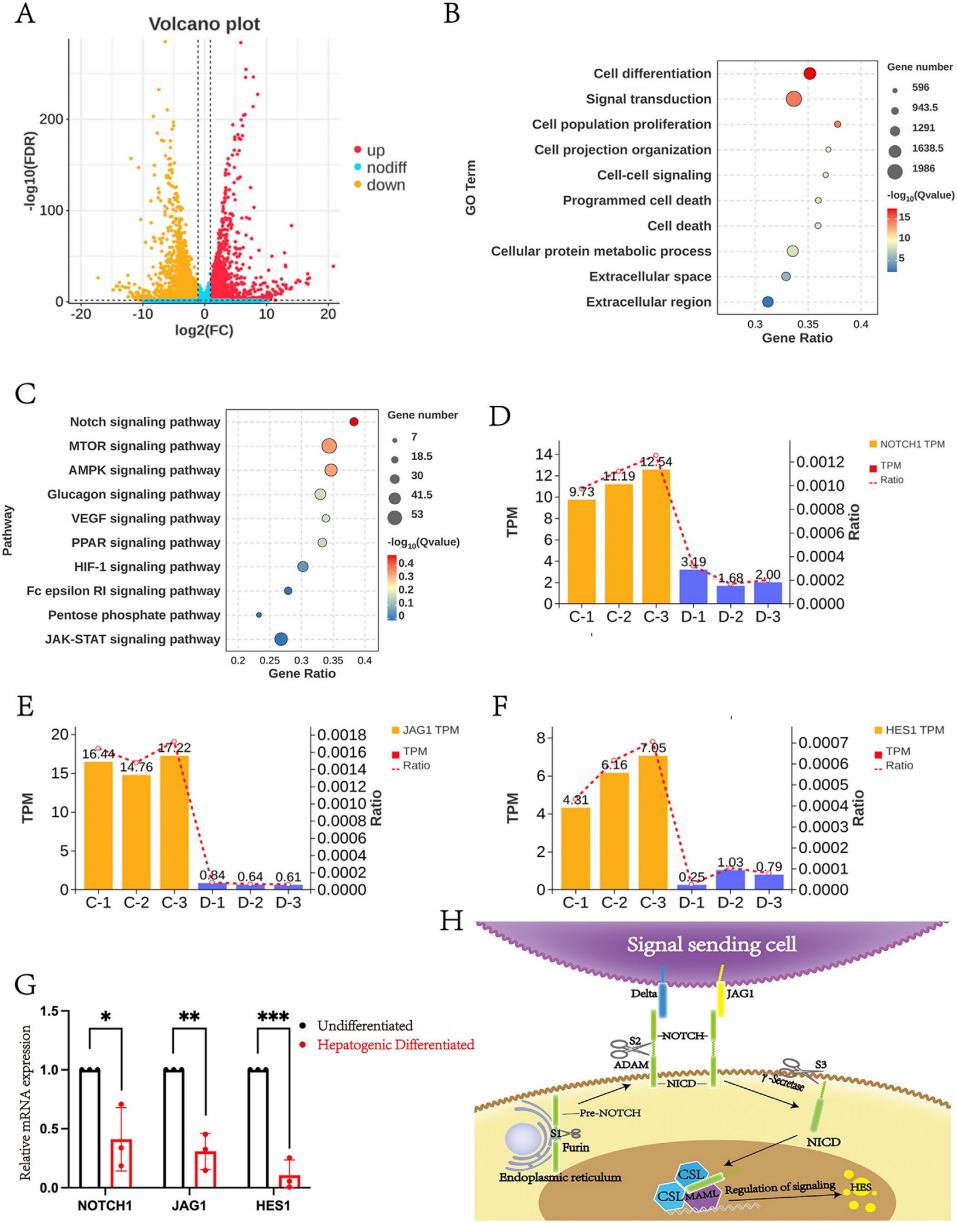
The RNA‐seq results confirmed that the Notch signalling pathway is involved in the hepatogenic differentiation process of MSCs. (A) Volcano plot showing DEGs between the Un‐Diff group and the Hep‐Diff group. (B) Bubble plot of GO analysis for both the Un‐Diff group and the Hep‐Diff group. (C) KEGG analysis reveals that the Notch signalling pathways is associated with cellular signal transduction functions. (D) Expression levels of the Notch signalling pathway receptor NOTCH1 in both the Un‐Diff group and the Hep‐Diff group. C, Un‐Diff group; D, Hep‐Diff group; TPM, transcripts per million reads; Ratio, ratio of genes to total differential genes. (E) Expression levels of the Notch signalling pathway ligand JAG1 in both the Un‐Diff group and the Hep‐Diff group. C, Un‐Diff group; D, Hep‐Diff group; TPM, Transcripts per million reads; Ratio, Ratio of genes to total differential genes. (F) Expression levels of the Notch signalling pathway downstream factor HES1 in both the Un‐Diff group and the Hep‐Diff group. C, Un‐Diff group; D, Hep‐Diff group; TPM, transcripts per million reads; Ratio, ratio of genes to total differential genes. (G) RT‐qPCR detection of the relative mRNA expression levels of Notch signalling pathway factors (NOTCH1, JAG1 and HES1) in both the Un‐Diff group and the Hep‐Diff group. *p < 0.05, **p < 0.01, ***p < 0.001. (H) Diagram of the Notch signalling pathway.

Several studies have demonstrated that the Notch signalling pathway can regulate the differentiation of MSCs [29, 30, 31]. To investigate the role of the Notch signalling pathway in hepatogenic differentiation, we performed RNA‐seq analysis on cells before and after differentiation. The results indicated significant differences in gene expression between MSCs and HLCs (Figure 2A). GO and Kyoto Encyclopaedia of Genes and Genomes (KEGG) analyses were conducted on DEGs, with GO analysis suggesting that the Notch signalling pathway was mainly involved in key intercellular signalling processes (Figure 2B,C). In addition, we observed that the expression levels of Notch receptor NOTCH1, its ligand JAG1 and the downstream factor HES1 were significantly reduced after hepatogenic differentiation (Figure 2D–F). The reliability of these DEG expression changes was further validated by qPCR (Figure 2G). NOTCH1, as an upstream regulator of the Notch signalling pathway, plays a crucial role in both activating and inhibiting this pathway. After binding to its ligand and undergoing cleavage by γ‐secretase, the NOTCH1 intracellular domain (NICD) subsequently translocates to the nucleus, where it mediates signal transduction by regulating the downstream factor HES1 (Figure 2H).

### 3.3 The Notch Signalling Pathway Regulates the Differentiation of MSCs

**FIGURE 3:**
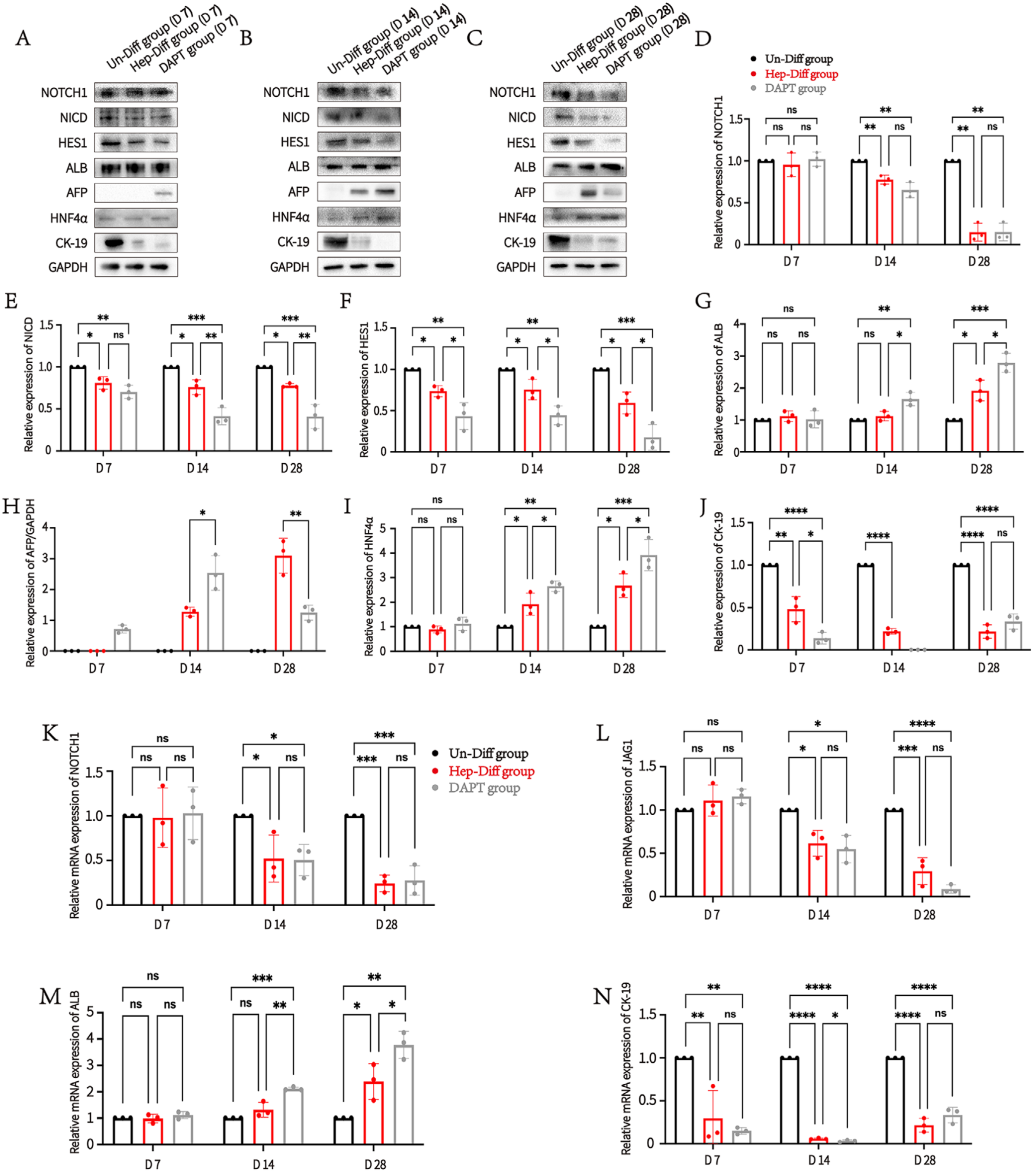
The Notch signalling pathway regulates the differentiation of MSCs into HLCs. Western blotting and RT‐qPCR were employed to assess the expression differences of various factors in the Un‐Diff, Hep‐Diff and DAPT groups. (A) Protein expression of Notch signalling pathway factors (NOTCH1, NICD and HES1) and hepatocyte‐associated factors (ALB, AFP, HNF4α and CK‐19) was detected by Western blotting in each group at 7 days (D 7). (B) Protein expression of Notch signalling pathway factors (NOTCH1, NICD and HES1) and hepatocyte‐associated factors (ALB, AFP, HNF4α and CK‐19) was detected by Western blotting in each group at 14 days (D 14). (C) Protein expression of Notch signalling pathway factors (NOTCH1, NICD and HES1) and hepatocyte‐associated factors (ALB, AFP, HNF4α and CK‐19) was detected by Western blotting in each group at 28 days (D 28). (D) Changes in the relative protein expression of NOTCH1 in each group at differentiation time points of D 7, D 14 and D 28. **p < 0.01. (E) Changes in the relative protein expression of NICD in each group at differentiation time points of D 7, D 14 and D 28. *p < 0.05, **p < 0.01, ***p < 0.001. (F) Changes in the relative protein expression of HES1 in each group at differentiation time points at D 7, D 14 and D 28. *p < 0.05, **p < 0.01, ***p < 0.001. (G) Changes in the relative protein expression of ALB in each group at differentiation time points of D 7, D 14 and D 28. *p < 0.05, **p < 0.01, ***p < 0.001. (H) Changes in the relative protein expression of AFP in each group at differentiation time points of D 7, D 14 and D 28. *p < 0.05, **p < 0.01, ***p < 0.001. (I) Changes in the relative protein expression of HNF4α in each group at differentiation time points of D 7, D 14 and D 28. *p < 0.05, **p < 0.01, ***p < 0.001. (J) Changes in the relative protein expression of CK‐19 in each group at differentiation time points of D 7, D 14 and D 28. *p < 0.05, ***p < 0.001, ****p < 0.0001. (K) Changes in the relative mRNA expression of NOTCH1 in each group at differentiation time points of D 7, D 14 and D 28. *p < 0.05, ***p < 0.001. (L) Changes in the relative mRNA expression of JAG1 in each group at differentiation time points of D 7, D 14 and D 28. *p < 0.05, ***p < 0.001, ****p < 0.0001. (M) Changes in the relative mRNA expression of ALB in each group at differentiation time points of D 7, D 14 and D 28. *p < 0.05, **p < 0.001, ***p < 0.001. (N) Changes in the relative mRNA expression of CK‐19 in each group at differentiation time points of D 7, D 14 and D 28. *p < 0.05, **p < 0.01, ****p < 0.0001.

We divided the cells into three groups: the Un‐Diff group, the Hep‐Diff group and the DAPT group to further validate the effect of the Notch signalling pathway on hepatogenic differentiation. DAPT has been shown to induce cell cycle arrest across various cell types, potentially affecting differentiation processes [32, 33]. To explore the effects of DAPT on cell cycle dynamics during hepatogenic differentiation of MSCs, cell cycle analysis was performed on three distinct groups of cells. The results indicated an increase in the G0/G1 phase and a decrease in the S phase, with no statistically significant changes in the G2/M phase between the Hep‐Diff and the DAPT‐treated group compared to the Un‐Diff. Moreover, no statistically significant differences in cell cycle progression were observed between the Hep‐Diff and DAPT groups (Figure S1A–D). In addition, a concentration of 10 μM was selected as the DAPT concentration, and cytotoxicity assays demonstrated that this concentration was non‐toxic to cells (Figure S2A). Subsequently, Proteins were extracted from these differentiated cells at 7, 14 and 28 days, and compared with proteins from the Un‐Diff group (Figure 3A–C). Western blotting analysis revealed no significant difference in the expression of the Notch signalling pathway receptor NOTCH1 among the three groups after 7 days of cell induction. However, with further differentiation, NOTCH1 protein expression was significantly reduced in both the Hep‐Diff group and the DAPT group compared to the Un‐Diff group (Figure 3D). Interestingly, there was no statistically significant difference in NOTCH1 protein expression between the Hep‐Diff group and the DAPT group throughout the differentiation process. This is primarily because DAPT inhibits γ‐secretase, preventing cleavage at the S3 site (Figure 2H), and therefore does not affect the full‐length expression of NOTCH1. This assumption was verified by the differences in NICD protein expression among the three groups (Figure 3E). Under DAPT intervention, both NICD and its downstream factor HES1 exhibited further downregulation (Figure 3F). These results suggest that the Notch signalling pathway is gradually downregulated during hepatogenic differentiation, with DAPT further inhibiting its activation. We then examined hepatocyte‐associated proteins across the three groups during differentiation. The results revealed that ALB was expressed gradually, and compared to the Hep‐Diff group, the ALB protein expression level was significantly higher in the DAPT group (Figure 3G). The Un‐Diff group did not express AFP, but AFP protein expression was detected in the DAPT group after 7 days of hepatogenic differentiation induction. By 14 days, AFP expression was observed in both the Hep‐Diff and DAPT groups, though the AFP expression level in the DAPT group was higher than that in the Hep‐Diff group. However, when AFP was detected at 28 days, it was found that the protein level of AFP expression in the DAPT group was lower compared to the Hep‐Diff group. This decrease was attributed to the gradual decline in AFP expression as the HLCs matured [34] (Figure 3H). The mature HLCs exhibited positive expression of HNF4α and negative expression of CK‐19 [35, 36]. Results for HNF4α and CK‐19 further confirm that inhibition of the Notch signalling pathway enhances HLC maturation (Figure 3I,J). In addition, we measured the mRNA levels of the Notch receptor NOTCH1 and its ligand JAG1. The results showed that mRNA levels of NOTCH1 and JAG1 decreased gradually during hepatogenic differentiation, with DAPT having no significant impact on their mRNA expression (Figure 3K,L). Simultaneous measurement of the mRNA levels of ALB and CK‐19 revealed expression trends consistent with their protein levels (Figure 3M,N). Overall, these results indicate that DAPT did not impact the cell cycle of MSCs during hepatogenic differentiation. In addition, the Notch signalling pathway is involved in the differentiation of MSCs into hepatocytes, and its inhibition can further promote the differentiation of MSCs into mature HLCs.

### 3.4 Increased NO Production Promotes S‐Nitrosylation of the NOTCH1

**FIGURE 4:**
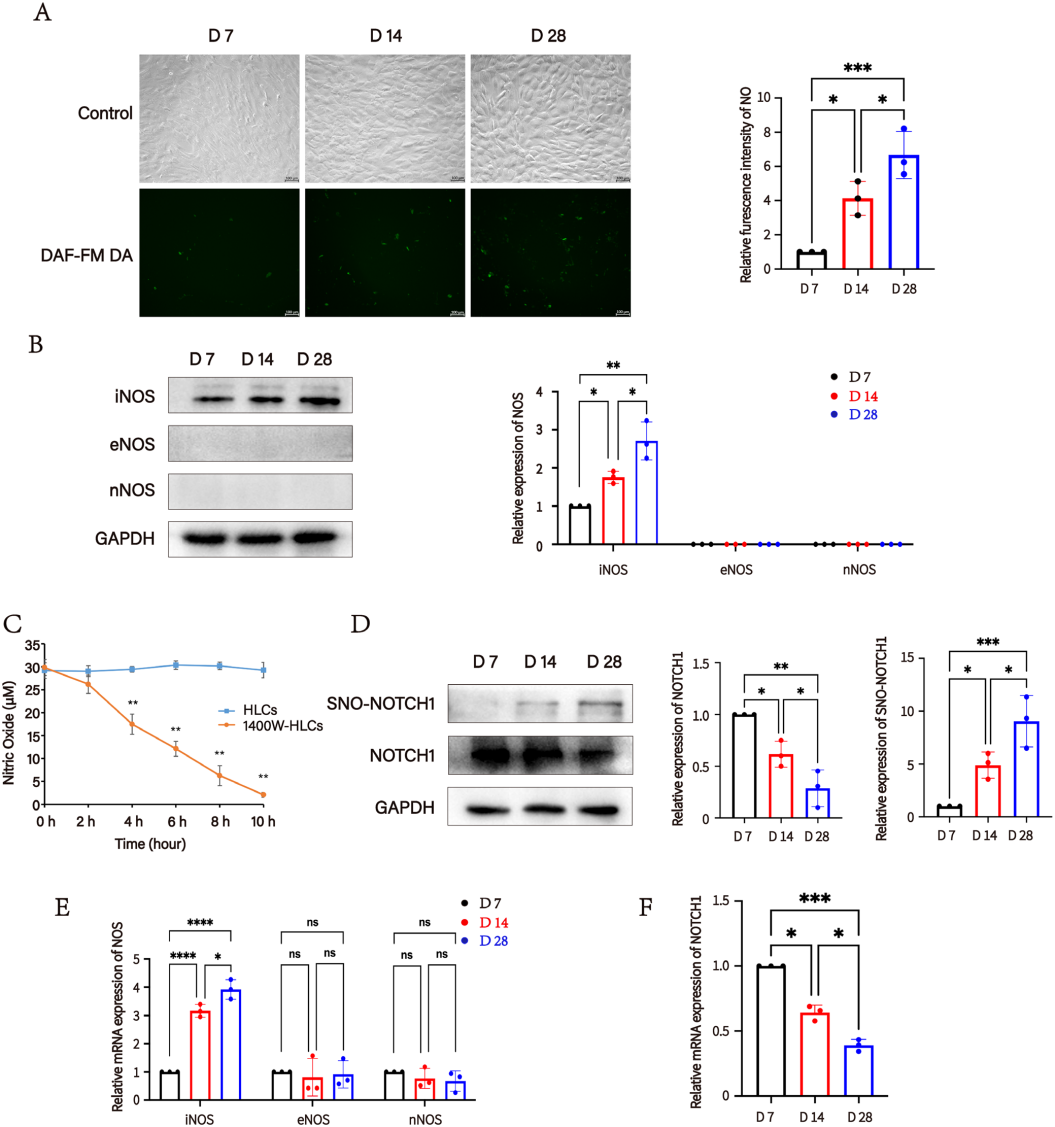
SNO‐NOTCH1 levels gradually increased while NOTCH1 protein levels gradually decreased during hepatogenic differentiation. (A) The fluorogram and relative expression of intracellular NO during hepatogenic differentiation for 7 days (D 7), 14 days (D 14) and 28 days (D 28). Scale bar = 100 μm. *p < 0.05, ***p < 0.001. (B) Relative protein expression of iNOS, eNOS and nNOS in cells undergoing hepatogenic differentiation for D 7, D 14 and D 28. *p < 0.05, **p < 0.01. (C) Effect of the selective iNOS inhibitor 1400 W on NO concentration in HLCs from 0 to 10 h. **p < 0.01. (D) Relative protein expression of NOTCH1 and relative expression of SNO‐NOTCH1 in MSCs undergoing hepatogenic differentiation for D 7, D 14 and D 28. *p < 0.05, **p < 0.01, ***p < 0.001. (E) Relative mRNA expression of iNOS, eNOS and nNOS in MSCs undergoing hepatogenic differentiation for D 7, D 14 and D 28. *p < 0.05, ****p < 0.0001. (F) Relative mRNA expression of NOTCH1 in MSCs undergoing hepatogenic differentiation for D 7, D 14 and D 28. *p < 0.05, ***p < 0.001.

To further investigate whether NO is involved in regulating the Notch signalling pathway‐mediated hepatogenic differentiation into HLCs, we employed the NO fluorescent probe DAF‐FM‐DA to measure NO content in MSCs that underwent hepatogenic differentiation for up to 7, 14, and 28 days. The results of the NO fluorescence intensity indicated a gradual increase in NO content during hepatogenic differentiation (Figure 4A). Endogenous NO is primarily synthesised by three subtypes of NOS: iNOS, eNOS and nNOS. We assessed the protein expression of these NOS subtypes and found that the increase in NO content was mainly attributed to the gradual expression of iNOS during hepatogenic differentiation, whereas eNOS and nNOS exhibited negative expression during this process (Figure 4B). To further verify that the increase in NO content was specifically related to iNOS expression, we used the selective iNOS inhibitor 1400 W. After treating HLCs with 1400 W, we observed whether the decrease in NO concentration was attributed to iNOS inhibition. The results demonstrated a marked reduction in NO levels in HLCs after 1400 W treatment (Figure 4C). To further elucidate the underlying mechanisms responsible for the observed increase in iNOS expression, MSCs were cultured for one week with the addition of a single inducing factor to the culture medium. The influence of each factor on iNOS expression levels was subsequently evaluated. The results indicated that the inducible factors, HGF and OSM, could significantly elevate iNOS expression (Figure S3). Moreover, high expression of NOS can elevate the levels of S‐nitrosylation [37]. Biotin switch assay findings showed a significant increase in the S‐nitrosylation degree of NOTCH1 during hepatogenic differentiation, while the protein level of NOTCH1 gradually decreased (Figure 4D). These outcomes were further supported by mRNA expression level data (Figure 4E,F).

### 3.5 SNO‐NOTCH1 Promotes Hepatogenic Differentiation Into HLCs by Inhibiting the Notch Signalling Pathway

**FIGURE 5:**
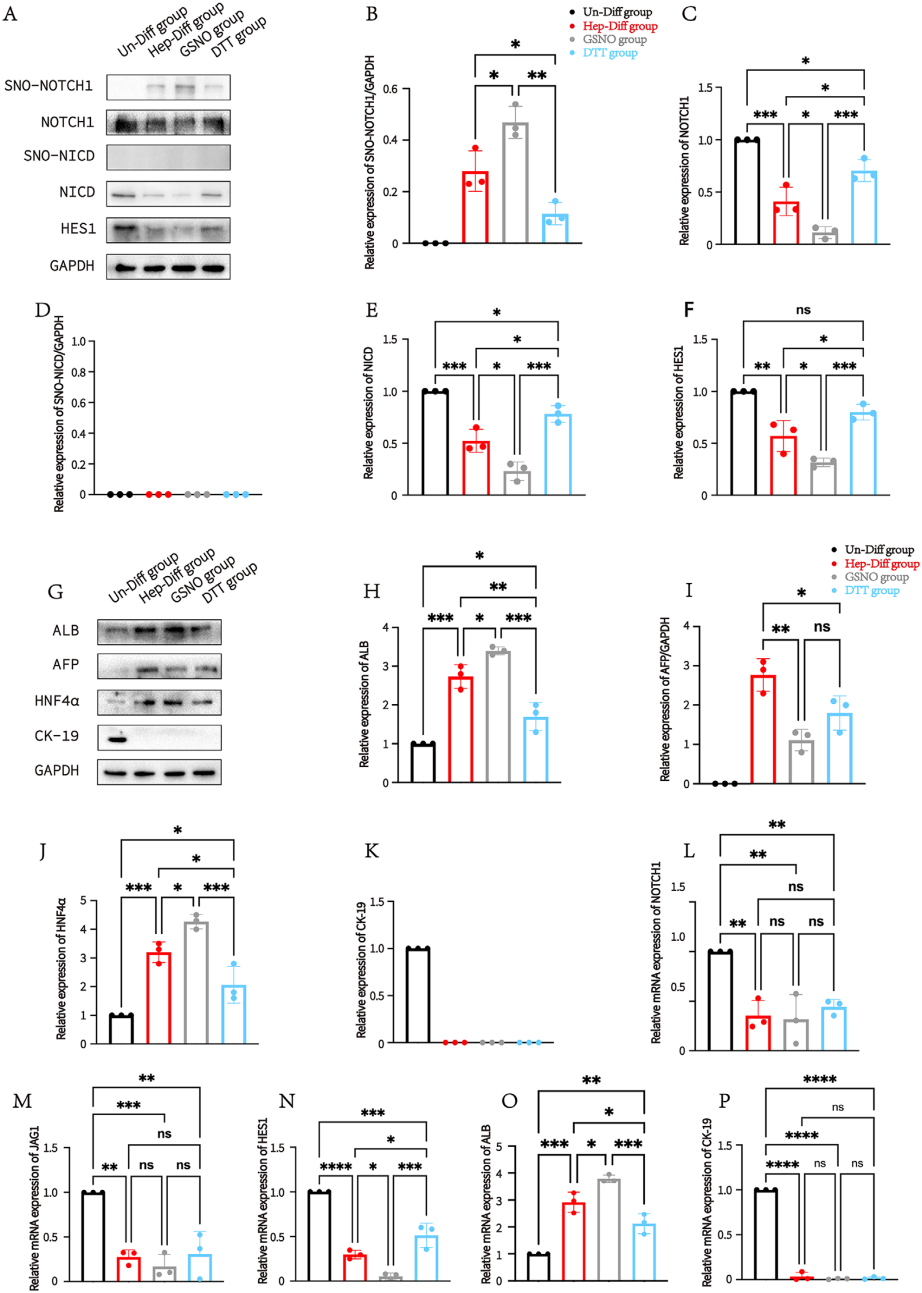
S‐nitrosylation of NOTCH1 promotes Notch signalling pathway‐mediated hepatogenic differentiation by inhibiting NOTCH1 self‐expression. (A) Hepatogenic differentiation was observed for up to 28 days. This includes protein expression levels of NOTCH signalling pathway factors (NOTCH1, NICD and HES1) and S‐nitrosylation levels of NOTCH1 and NICD in the Un‐Diff, Hep‐Diff, GSNO and DTT groups. (B) Differences in relative protein expression of SNO‐NOTCH1 among the four groups. *p < 0.05, **p < 0.01. (C) Differences in relative protein expression of NOTCH1 among the four groups. *p < 0.05, ***p < 0.001. (D) Differences in relative protein expression of SNO‐NICD among the four groups. *p < 0.05, ***p < 0.001. (E) Differences in relative protein expression of NICD among the four groups. (F) Differences in relative protein expression of HES1 among the four groups. *p < 0.05, **p < 0.01, ***p < 0.001. (G) Hepatogenic differentiation was assessed up to 28 days. This includes protein expression levels of hepatocyte‐associated factors (ALB, AFP, CK‐18 and CK‐19) in the Un‐Diff, Hep‐Diff, GSNO, and DTT groups. (H) Differences in relative protein expression of ALB among the four groups. *p < 0.05, **p < 0.01, ***p < 0.001. (I) Differences in relative protein expression of AFP among the four groups. *p < 0.05, **p < 0.01. (J) Differences in relative protein expression of HNF4α among the four groups. *p < 0.05, ***p < 0.001. (K) Differences in relative protein expression of CK‐19 among the four groups. (L) Differences in relative mRNA expression of NOTCH1 among the four groups. **p < 0.01. (M) Differences in relative mRNA expression of JAG1 among the four groups. **p < 0.01, ***p < 0.001. (N) Differences in relative mRNA expression of HES1 among the four groups. *p < 0.05, **p < 0.01, ***p < 0.001. (O) Differences in relative mRNA expression of ALB among the four groups. *p < 0.05, **p < 0.01, ***p < 0.001. (P) Differences in relative mRNA expression of CK‐19 among the four groups. ****p < 0.0001.

To further explore whether NO‐induced SNO‐NOTCH1 is involved in Notch signalling pathway‐mediated hepatogenic differentiation. During the induction process, the exogenous NO donor GSNO and the protein S‐nitrosylation modification inhibitor DTT were added to regulate the level of SNO‐NOTCH1. A concentration of 10 μM was selected for both GSNO and DTT (Figure S2B,C). The non‐toxicity of this concentration to cells was confirmed through cytotoxicity assays. The results of the Biotin Switch Assay showed high expression of SNO‐NOTCH1 at hepatogenic differentiation up to 28 days, with a further increase of SNO‐NOTCH1 in the GSNO group, while a decrease of SNO‐NOTCH1 in the DTT group (Figure 5A,B). Interestingly, SNO‐NOTCH1 was negatively correlated with NOTCH1, when SNO‐NOTCH1 was highly expressed, the level of NOTCH1 decreased and vice versa (Figure 5A–C). In addition, we examined the expression level of SNO‐NICD and NICD. The results of the biotin switch assay showed that SNO‐NICD was negatively detected during hepatogenic differentiation, and the NICD protein expression was consistent with the protein expression of NOTCH1 (Figure 5D,E). Thus, it can be predicted that the decrease of NICD was owing to the change in NOTCH1 self‐activation, and the main site of NOTCH1 S‐nitrosylation was located on the cellular extracellular receptor domain (NECD). The downstream factor of Notch signalling pathway, HES1, was similarly regulated by the expression of SNO‐NOTCH1 (Figure 5F). The above results suggest that SNO‐NOTCH1 is involved in regulating the activation of the Notch signalling pathway. We further explored the effect of changes in SNO‐NOTCH1 levels on hepatogenic differentiation (Figure 5G). The protein expression of ALB and HNF4α was higher in the GSNO group than that of the Hep‐Diff group, whereas the protein expression of ALB and HNF4α was lower in the DTT group than that of the Hep‐Diff group (Figure 5H,J). According to previous studies, AFP was lowly expressed in mature liver tissues; our results further showed that AFP levels were lower in the GSNO group than that of the Hep‐Diff group which indicated that exogenous NO donor GSNO could promote hepatogenic differentiation. However, the level of AFP in the DTT group was also lower than that of the Hep‐Diff group, which may be because DTT inhibits the differentiation process by suppressing SNO‐NOTCH1, leaving AFP expression at an early stage of hepatogenic differentiation (Figure 5I). CK‐19 was highly expressed in the Un‐Diff group, while it was negatively expressed in the Hep‐Diff, GSNO and DTT group (Figure 5K). In addition, we also examined the mRNA expression levels of Notch signalling pathway receptors NOTCH1, JAG1 and HES1 (Figure 5L–N), as well as the expression levels of hepatocyte‐related genes (Figure 5O,P). SNO‐NOTCH1 did not affect the mRNA expression of NOTCH1 and JAG1, thus the mRNA expression of NOTCH1 and JAG1 were not statistically different in the Hep‐Diff, GSNO and DTT groups, but it affected the mRNA expression of the downstream factor HES1. The above results reveal that S‐nitrosylation of NOTCH1 promotes hepatogenic differentiation of MSCs.

## 4 Discussion

Transplantation of HLCs has been widely studied in recent years as a highly promising treatment that can replace hepatocyte and liver transplantation. Currently, stem cells that can be induced and differentiated into HLCs include embryonic stem cells (ESCs), induced pluripotent stem cells (iPSCs) and MSCs. However, the induced HLCs from ESCs do not fully match the phenotype of adult hepatocytes [38], while iPSC‐derived HLCs carry a risk of carcinogenesis owing to potential gene mutations during cell transplantation [39]. In contrast, MSCs are considered ideal for differentiating into HLCs because of their low immunogenicity and ease of availability [40].

Exploring the mechanisms underlying the hepatogenic differentiation of MSCs is crucial for advancing the clinical application of HLCs. In this study, we not only introduced a novel method to promote the differentiation of MSCs into HLCs but also revealed a new regulatory mechanism involved in the hepatogenic differentiation process of MSCs. Although the Notch signalling pathway has been identified as a regulator of MSC differentiation, its specific mechanisms remain not fully understood [41, 42]. Our experimental results demonstrate that HLCs differentiated from MSCs exhibit more mature hepatocyte‐related functions and characteristics following the inhibition of the Notch signalling pathway. This suggests that the Notch signalling pathway plays a pivotal role in regulating hepatogenic differentiation.

Recent studies have revealed that the nitrosylation modification of the protein cysteine sulfhydryl group by NO plays a crucial role in cell differentiation, cell fate determination, apoptosis, etc. NO is produced by three isoforms of NOS: nNOS, iNOS and eNOS, which induce proteins to undergo S‐nitrosylation [43]. In addition, NOTCH1, as the primary receptor of the Notch signalling pathway, is a key molecule in regulating this pathway and cell differentiation [44]. Previous research has demonstrated that NO can influence the expression of NOTCH1 [17, 18]. Our data indicated that endogenous NO levels progressively increase during hepatogenic differentiation, primarily as a result of the gradual upregulation of iNOS. To investigate the underlying causes of elevated iNOS expression, MSCs were cultured over a one‐week period with a variety of distinct inducing factors. Subsequently, the impact of each factor on iNOS expression levels was compared. It was determined that HGF and OSM may contribute to the increased iNOS expression observed during the hepatogenic differentiation of MSCs. To further investigate whether NO is involved in the formation of SNO‐NOTCH1 and regulates the Notch signalling pathway, we employed a biotin switch assay to assess SNO‐NOTCH1 levels. Our experimental results showed that SNO‐NOTCH1 remains highly expressed throughout hepatogenic differentiation, while the expression of NOTCH1 decreases gradually.

**FIGURE 6:**
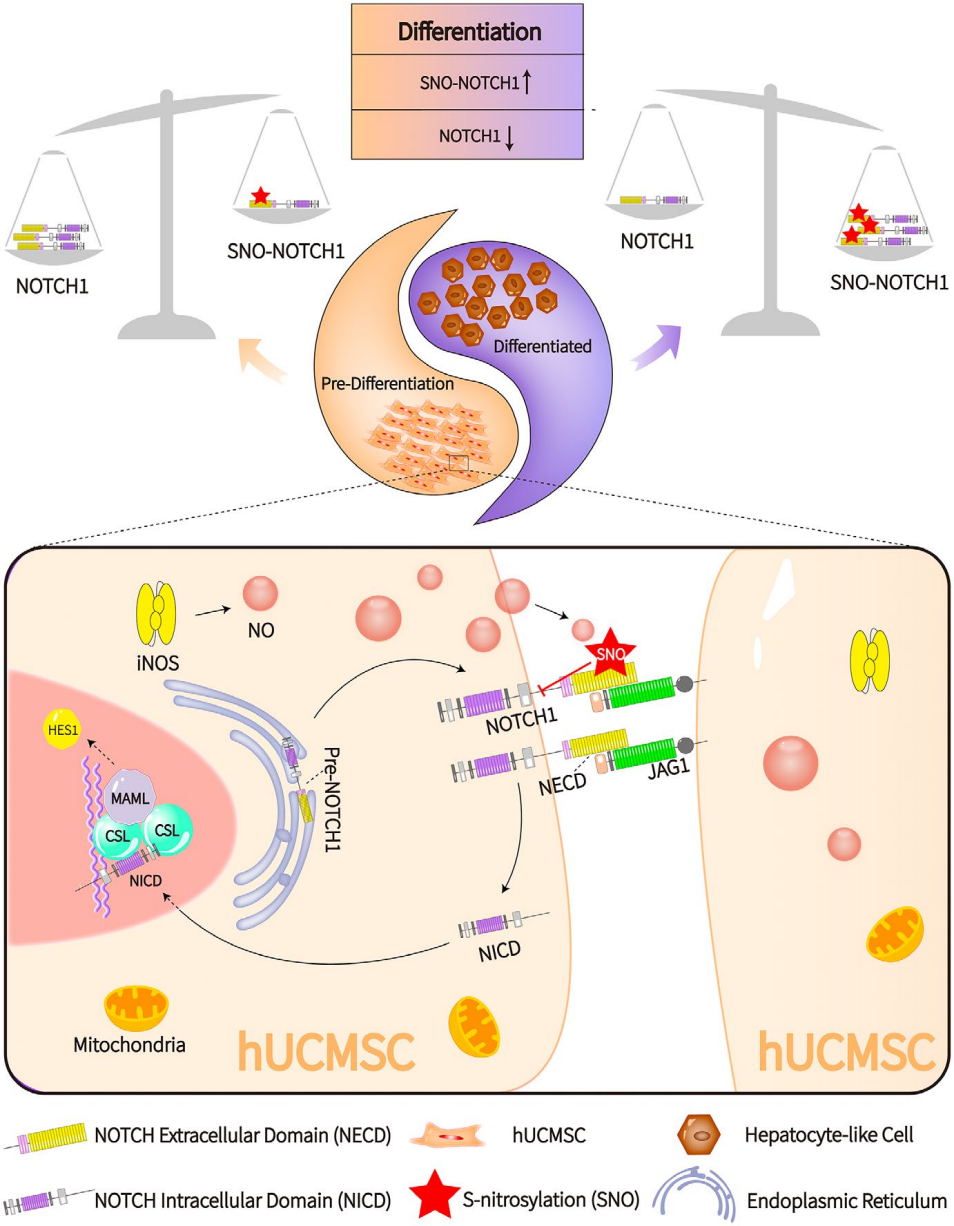
The schematic diagram illustrates that the increase in NO content during the differentiation of MSCs into HLCs is due to the high expression of iNOS and that NO causes S‐nitrosative modification of the extracellular domain of NECD, which leads to the reduction in the expression level of NOTCH1, thereby inhibiting the Notch signalling pathway. Inhibition of the Notch signalling pathway promotes the process of hepatogenic differentiation. During the whole differentiation process, due to the presence of NO, SNO‐NOTCH1 and NOTCH1 are like in a Yin and Yang relationship, but closely linked.

Next, we added exogenous NO donor GSNO and exogenous S‐nitrosylation inhibitor DTT during the induction process and differentiation process, respectively, to investigate whether SNO‐NOTCH1 regulates the Notch signalling pathway. The results confirmed that when DTT inhibits SNO‐NOTCH1 levels, the inhibitory effect of SNO‐NOTCH1 on the Notch signalling pathway is counteracted. Conversely, an increase in SNO‐NOTCH1 levels further enhances the inhibition of the Notch signalling pathway. Notably, elevated SNO‐NOTCH1 levels correlate with mRNA and protein expressions related to hepatocytes in HLCs becoming more similar to those of mature hepatocytes. In contrast, the downregulation of SNO‐NOTCH1 impedes hepatogenic differentiation. In addition, our experimental results unveiled that the S‐nitrosylation modification site on NOTCH1 is located in the extracellular domain of the protein, while the expression of NICD is not influenced by NOTCH1 S‐nitrosylation modification (Figure 6).

The NECD region contains three cysteine‐rich Lin12‐Notch repeat (LNR) sequences. These sequences are crucial for regulating the activation of the Notch signalling pathway and also serve as a basis for biochemical modifications such as S‐nitrosylation [45]. Furthermore, we utilised a specialised nitrosylation site prediction website (http://sno.biocuckoo.org) to predict specific amino acid residues in the extracellular LNR structural domain of the Notch receptor that may undergo nitrosylation. The residues identified included Cys6, Cys15 and Cys19, among others. However, further in‐depth research is necessary to confirm whether the S‐nitrosylation of NOTCH1 specifically targets the cysteine residues within these LNR sequences. Although our research suggests that S‐nitrosylation acting on NOTCH1 could enhance the directed differentiation of MSCs into HLCs, previous studies have shown that NO‐mediated S‐nitrosylation plays an inhibitory role in the differentiation of MSCs into vascular endothelial cells [46]. Another related study demonstrated that in the absence of GSNO reductase, a denitrosylase that regulates protein S‐nitrosylation, MSCs exhibited decreased adipogenesis but increased osteoblastogenesis. This effect was primarily mediated by the regulation of SNO‐PPARγ levels [47]. Therefore, NO‐mediated protein S‐nitrosylation does not operate as a fixed, unchanging mechanism but rather as a flexible regulatory mode that can promote the specific induction and differentiation of MSCs. Consequently, when studying the multifunctional role of S‐nitrosylation in the differentiation of MSCs into other tissue target cells, specific research results should be consulted.

The acquisition of HLCs requires a substantial initial cell source to ensure large‐scale generation, which is crucial for the clinical treatment of liver diseases. This presents a major challenge for the clinical translation of HLCs [48, 49, 50]. Current methods for harvesting HLCs include cell co‐culture [51], cytokine induction [52] and transgene induction [53], but none have proven effective in producing an adequate quantity of immature HLCs. Our previous study indicated that 3D‐cultured HLCs exhibit greater maturity compared to those cultured in 2D [34]. However, regardless of the culture method used, it is important to explore the regulatory mechanisms involved in hepatogenic differentiation. This will provide an important theoretical foundation and experimental data necessary for the large‐scale preparation of mature HLCs, which is pivotal for future treatments of end‐stage liver disease.

Although our research has yielded some relatively meaningful conclusions, several limitations need to be acknowledged. Firstly, while the Notch signalling pathway is known to play an important regulatory role in the differentiation of MSCs, we did not explore other molecules involved in regulating this pathway. The activation of the Notch signalling pathway is complex, including multiple regulatory molecules and interactions with other signalling pathways [54, 55, 56]. Secondly, recent years have seen the emergence of new protein S‐nitrosylation detection methods, such as biotin switch assay with isotope‐coded affinity tags or liquid chromatography–tandem mass spectrometry [57, 58]. These advanced techniques offer improved accuracy in identifying target proteins undergoing S‐nitrosylation and in detecting the expression of S‐nitrosylated proteins. Since the biotin switch assay can already detect results for differences in protein S‐nitrosylation expression, we only used this method to analyse S‐nitrosative modifications of proteins. Thirdly, while it was previously believed that protein S‐nitrosylation resulted from non‐enzymatic catalysis by free intracellular NO as well as certain SNO‐like molecules (such as SNO‐CoA), a recent study suggests that recombinant biliverdin reductase B (BLVRB), a liver‐expressed enzyme involved in bilirubin production, may also act as an SNO‐modifying enzyme [59]. Further investigation is needed to determine whether BLVRB catalyses the S‐nitrosylation of NOTCH1 during the hepatogenic differentiation of MSCs and to clarify its specific role in this process. Finally, NO, as a signalling molecule, is ubiquitously present in cells and tissues, with the formation of SNO‐NOTCH1 being mediated by NO. The potential off‐target effects of elevated NO levels during the differentiation of MSCs have not been extensively studied. This aspect was not addressed in this study due to the complex mechanisms involved. Future investigations will further explore this topic.

## 5 Conclusion

In conclusion, these results demonstrate that high expression of iNOS during hepatogenic differentiation leads to elevated NO levels. This, in turn, leads to the S‐nitrosylation of NOTCH1. The SNO‐NOTCH1 modification contributes to the inhibition of the Notch signalling pathway, thereby promoting the hepatogenic differentiation of MSCs into HLCs.

## Author Contributions

**Xuesong Wang:** conceptualization (supporting), data curation (lead), formal analysis (lead), writing – original draft (lead), writing – review and editing (supporting). **Yan Xu:** data curation (supporting), formal analysis (supporting), writing – original draft (supporting). **Yue Wang:** data curation (supporting), formal analysis (supporting), writing – original draft (supporting). **Xingkun Tang:** formal analysis (supporting), software (supporting). **Xiaolei Zhou:** formal analysis (supporting), software (lead). **Wenming Lu:** data curation (supporting), formal analysis (supporting). **Wenjie Chen:** data curation (equal), formal analysis (equal), funding acquisition (lead). **Lincai Li:** formal analysis (supporting), software (supporting). **Lin Zhou:** formal analysis (equal), writing – review and editing (equal). **Junsong Ye:** conceptualization (lead), funding acquisition (lead), writing – review and editing (lead).

## Ethics Statement

The study protocol was approved by the Ethics Committee of Gannan Medical University (protocol code 2020056).

## Consent

There are no human subjects in this article and informed consent is not applicable.

## Conflicts of Interest

The authors declare no conflicts of interest.

## Supporting information


**Figure S1.** Cell cycle analysis. Un‐Diff group, The MSCs reached 80% confluence; Hep‐Diff group, MSCs of hepatogenic differentiation for 24 h; DAPT group, DAPT was added to MSCs of hepatogenic differentiation for 24 h. (A) The results of the cell cycle analysis in the Un‐Diff group. (B) The results of the cell cycle analysis in the Hep‐Diff group. (C) The results of the cell cycle analysis in the DAPT group. (D) Cell cycle expression in G0/G1 phase, S phase and G2/M for each group. ****p < 0.0001.

**Figure S2.** Cytotoxicity assay. (A) Detection of cell viability after treatment with different concentrations of DAPT. ****p < 0.0001. (B) Detection of cell viability after treatment with different concentrations of GSNO. *p < 0.01. (C) Detection of cell viability after treatment with different concentrations of DTT. *p < 0.01, ***p < 0.001.

**Figure S3.** Differences in relative protein expression of iNOS following a one‐week incubation period of MSCs with a single inducing factor.

**Table S1.** Sequences of primers for RT‐PCR.

## Data Availability

The raw data supporting the conclusions of this article will be made available by the authors on request.
