# Supplementary figures and images for: S‐Nitrosylation of NOTCH1 Regulates Mesenchymal Stem Cells Differentiation Into Hepatocyte‐Like Cells by Inhibiting Notch Signalling Pathway
Source: J Cell Mol Med. 2024 Dec 10;28(23):e70274. doi: 10.1111/jcmm.70274 (PMC11629812; doi:10.1111/jcmm.70274)

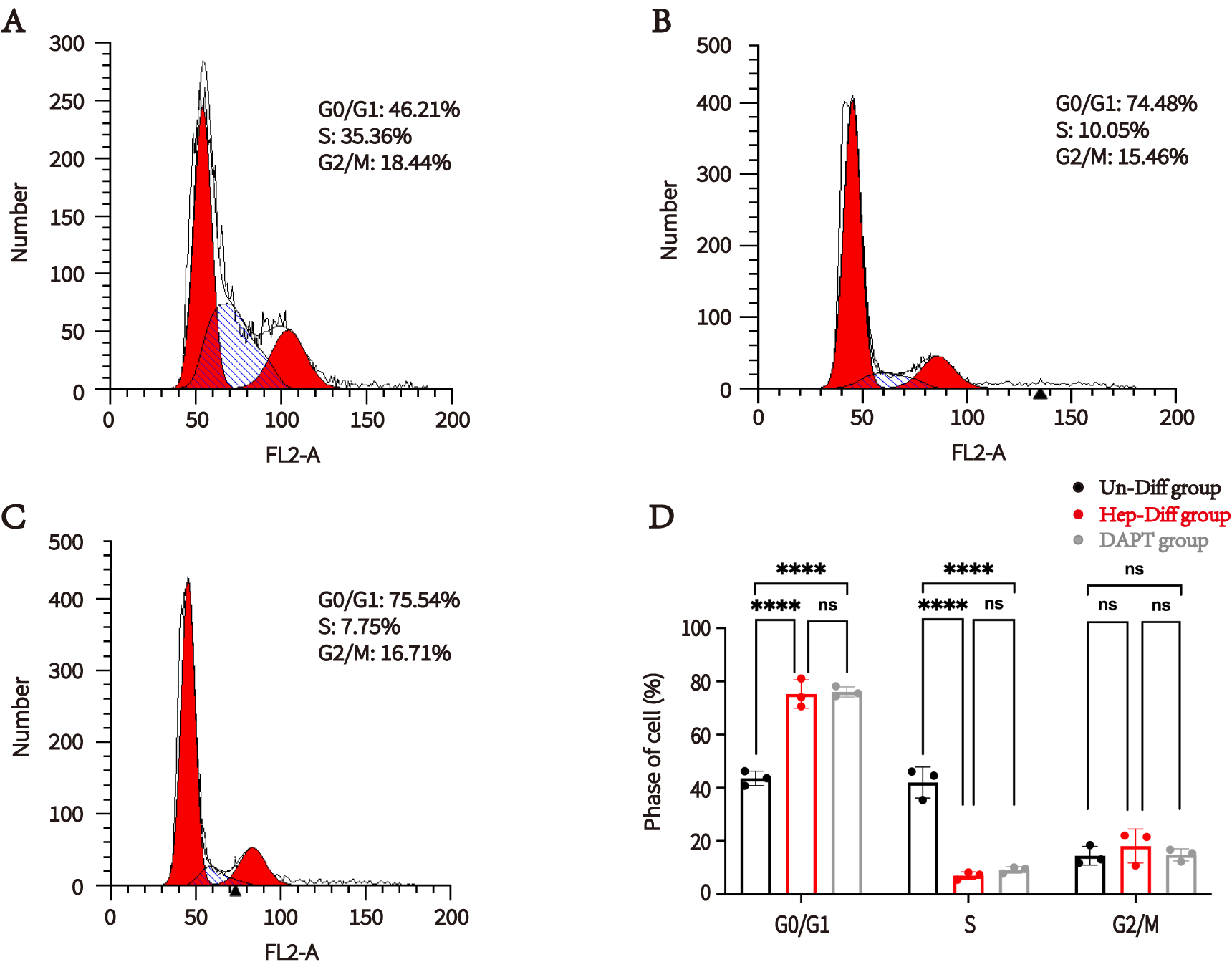

Supplement: Supplementary file 1 — Figure S1. Cell cycle analysis. Un‐Diff group, The MSCs reached 80% confluence; Hep‐Diff group, MSCs of hepatogenic differentiation for 24 h; DAPT group, DAPT was added to MSCs of hepatogenic differentiation for 24 h. (A) The results of the cell cycle analysis in the Un‐Diff group. (B) The results of the cell cycle analysis in the Hep‐Diff group. (C) The results of the cell cycle analysis in the DAPT group. (D) Cell cycle expression in G0/G1 phase, S phase and G2/M for each group. ****p < 0.0001. [file JCMM-28-e70274-s004.tif]

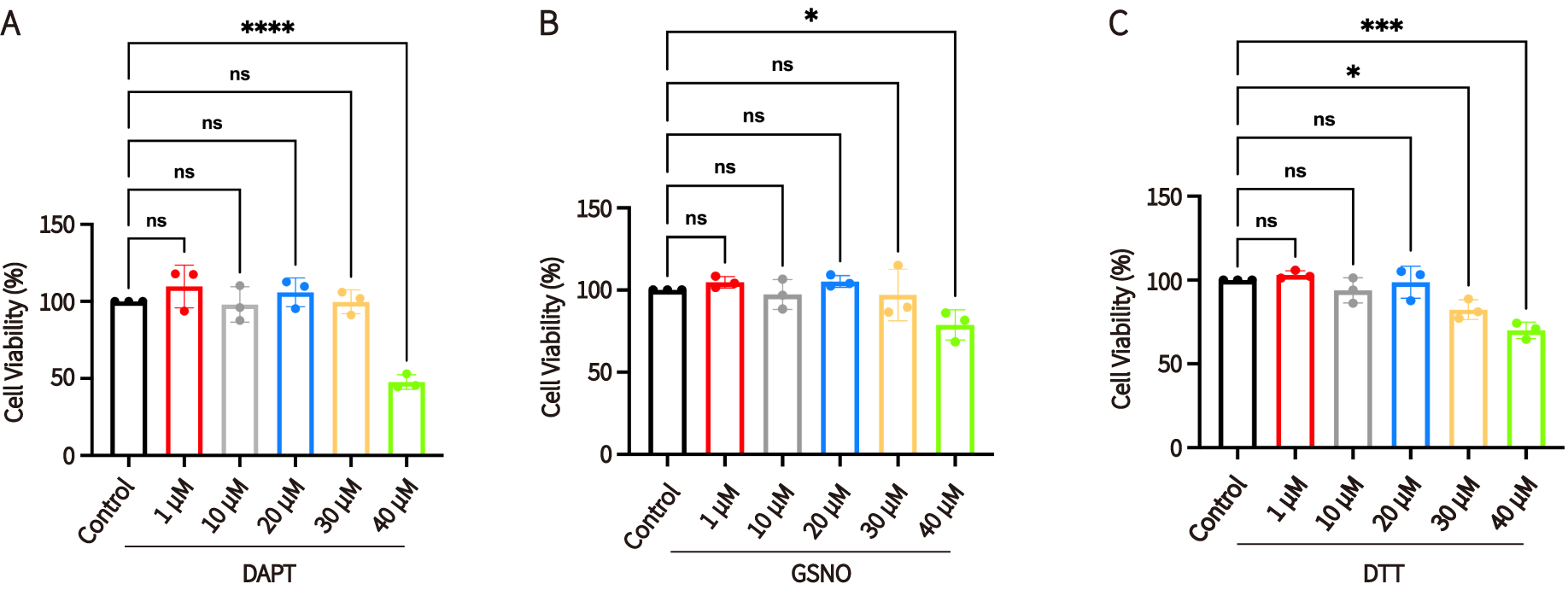

Supplement: Supplementary file 2 — Figure S2. Cytotoxicity assay. (A) Detection of cell viability after treatment with different concentrations of DAPT. ****p < 0.0001. (B) Detection of cell viability after treatment with different concentrations of GSNO. *p < 0.01. (C) Detection of cell viability after treatment with different concentrations of DTT. *p < 0.01, ***p < 0.001. [file JCMM-28-e70274-s003.tif]

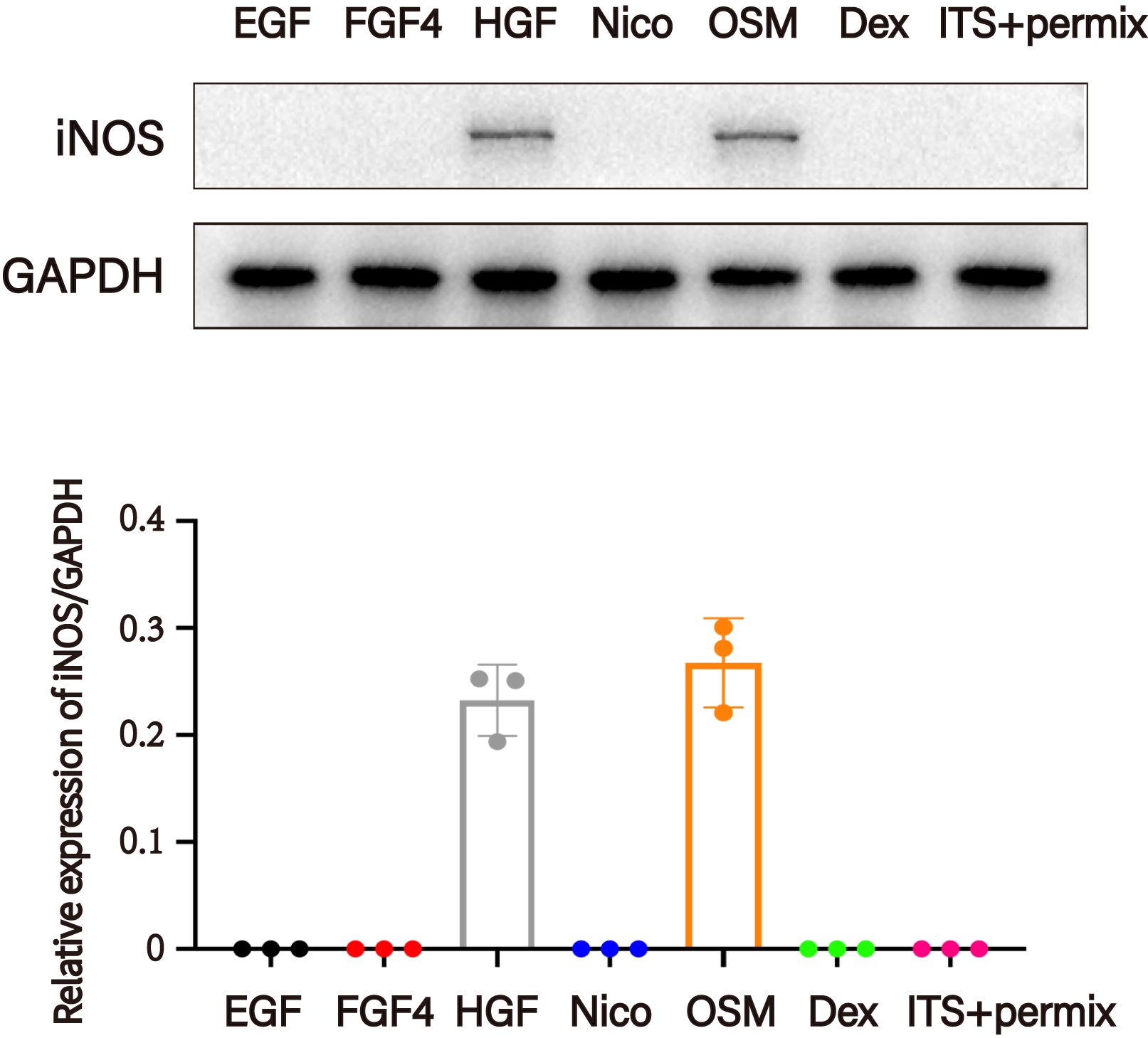

Supplement: Supplementary file 3 — Figure S3. Differences in relative protein expression of iNOS following a one‐week incubation period of MSCs with a single inducing factor. [file JCMM-28-e70274-s002.tif]
