# Supplementary material for: S‐Nitrosylation of NOTCH1 Regulates Mesenchymal Stem Cells Differentiation Into Hepatocyte‐Like Cells by Inhibiting Notch Signalling Pathway
Source: J Cell Mol Med. 2024 Dec 10;28(23):e70274. doi: 10.1111/jcmm.70274 (PMC11629812; doi:10.1111/jcmm.70274)
Supplement: Supplementary file 4 — Table S1. Sequences of primers for RT‐PCR. [file JCMM-28-e70274-s001.docx]

**Supplementary File 1. Sequences of primers for RT-PCR**

| Name | Forward | Reverse |
| --- | --- | --- |
| NANOG | CCCCAGCCTTTACTCTTCCTA | CCAGGTTGAATTGTTCCAGGTC |
| SOX2 | ATCAGGAGTTGTCAAGGCAGAG | AGAGGCAAACTGGAATCAGGA |
| OCT4 | AAGCGATCAAGCAGCGAC | GGAAAGGGACCGAGGAGTA |
| ALB | TGCAACTCTTCGTGAAACCTATG | ACATCAACCTCTGGTCTCACC |
| AFP | GCTTGGTGGTGGATGAAACA | TCCTCTGTTATTTGTGGCTTTTG |
| HNF4α | TCCATACGCATCCTTGACGA | TGATGGCTTTGAGGTAGGCA |
| CK-18 | GATATCCGTGTCCCGCTCTG | CACCTTGTCTAGGTAGCTGGC |
| CK-19 | AGGAGATTGCCACCTACCG | CCTTCCCATCCCTCTACCC |
| NOTCH1 | ATGCAGAACAACAGGGAGGA | ACCAGGTTGTACTCGTCCAG |
| JAG1 | CACGCGTCATTGTGTTACCT | TCCCGGCTTTCTTTCCTTCT |
| HES1 | CATTCCAAGCTGGAGAAGGC | GTCACCTCGTTCATGCACTC |
| iNOS | TCATCCGCTATGCTGGCTAC | CCCGAAACCACTCGTATTTGG |
| eNOS | ATGCTCCCACCAGGGCATCA | GTCCTTGAGTCTGACATTAGGG |
| nNOS | CCTCCCGCCCTGCACCATCTT | CTTGCCCCATTTCCATTCCTCGTA |
| GAPDH | AGCCACATCGCTCAGACAC | GCCCAATACGACCAAATCC |
